# Antibodies to the inositol 1,4,5-trisphosphate receptor type 1 (ITPR1) in cerebellar ataxia

**DOI:** 10.1186/s12974-014-0206-3

**Published:** 2014-12-11

**Authors:** Sven Jarius, Madeleine Scharf, Nora Begemann, Winfried Stöcker, Christian Probst, Irina I Serysheva, Sigrun Nagel, Francesc Graus, Dimitri Psimaras, Brigitte Wildemann, Lars Komorowski

**Affiliations:** Molecular Neuroimmunology, Department of Neurology, University of Heidelberg, Im Neuenheimer Feld 400, 69120 Heidelberg, Germany; Institute of Experimental Immunology, affiliated to Euroimmun AG, Seekamp 31, 23560 Lübeck, Germany; Department of Biochemistry and Molecular Biology, The University of Texas Medical School at Houston, 6431 Fannin Street, Houston, TX 77030 USA; Leibniz Institute for Age Research/Fritz Lipmann Institute, Beutenbergstraße 11, D-07745 Jena, Germany; Institut d’ Investigació Biomèdica August Pi i Sunyer (IDIBAPS), Villarroel 170, Barcelona, 08036 Spain; Department of Neurology Mazarin, Hôpital Pitié-Salpêtrière, University René Descartes, 47-83, Boulevard de l’Hôpital, 75651 Paris, Cedex 13 France

**Keywords:** Anti-Ca, Anti-Sj, Autoantibodies, Autoimmune cerebellar ataxia, Inositol 1,4,5-trisphosphate receptor type 1 (ITPR1, IP3RI, IP3R1, INSP3R1, ACV, CLA4, PPP1R94, SCA15, SCA29), Purkinje cells, Rho GTPase activating protein 26 (ARHGAP26, GTPase regulator associated with focal adhesion kinase pp125, GRAF, GRAF1, Oligophrenin-1-like protein, OPHN1L)

## Abstract

We report on a serum autoantibody associated with cerebellar ataxia. Immunohistochemical studies of sera from four patients referred for autoantibody testing revealed binding of high-titer (up to 1:5,000) IgG antibodies, mainly IgG1, to the molecular layer, Purkinje cell layer, and white matter on mouse, rat, porcine, and monkey cerebellum sections. The antibody bound to PC somata, dendrites, and axons, resulting in a binding pattern similar to that reported for anti-Ca/anti-ARHGAP26, but did not react with recombinant ARHGAP26. Extensive control studies were performed to rule out a broad panel of previously described paraneoplastic and non-paraneoplastic anti-neural autoantibodies. The characteristic binding pattern as well as double staining experiments suggested inositol 1,4,5-trisphosphate receptor type 1 (ITPR1) as the target antigen. Verification of the antigen included specific neutralization of the tissue reaction following preadsorption with ITPR1 (but not ARHGAP26) and a dot-blot assay with purified ITPR1 protein. By contrast, anti-ARHGAP26-positive sera did not bind to ITPR1. In a parallel approach, a combination of histoimmunoprecipitation and mass spectrometry also identified ITPR1 as the target antigen. Finally, a recombinant cell-based immunofluorescence assay using HEK293 cells expressing ITPR1 and ARHGAP26, respectively, confirmed the identification of ITPR1. Mutations of ITPR1 have previously been implicated in spinocerebellar ataxia with and without cognitive decline. Our findings suggest a role of autoimmunity against ITPR1 in the pathogenesis of autoimmune cerebellitis and extend the panel of diagnostic markers for this disease.

## Background

Autoimmune cerebellar ataxia (ACA) is an etiologically and pathologically heterogeneous syndrome. Besides multiple sclerosis, paraneoplastic neurological disorders are the most common cause of ACA [1,2]. Many cases of paraneoplastic ACA are associated with serum or cerebrospinal fluid autoantibodies to neuronal and/or glial antigens such as anti-Hu [3], anti-Ri [4], anti-Yo [5], anti-CV2/CRMP5 [6,7], PCA-Tr/anti-DNER [8], anti-Zic4 [9], anti-protein kinase C gamma (PKCγ) [10], anti-mGluR1 [11,12], PCA2 [13], ANNA3 [14], CARPVIII [15,16], or anti-voltage-gated calcium channels (VGCC) [17]. In patients with non-paraneoplastic ACA, antibodies to tissue transglutaminase [18,19], glutamate receptor δ2 (GluRδ2) [20,21], and Homer-3 [22] have been described. Some antibodies have been reported both in paraneoplastic and in non-paraneoplastic contexts, such as antibodies to GABA B receptors (GABABR), glutamate decarboxylase (GAD) [23-27], or dipeptidyl-peptidase 6 (DPPX) [28,29].

Recently, we identified a novel Purkinje cell (PC) autoantibody (anti-Ca) in patients with autoimmune cerebellar ataxia that targets the RhoGTPase-activating protein 26 (ARHGAP26, GRAF) [30-32]. Here, we report an autoantibody that binds to PC somata, dendrites, and axons in a pattern almost identical to that of anti-Ca/anti-ARHGAP26 [30-32] but targets the inositol 1,4,5-trisphophate receptor type 1 (ITPR1). The antibody was found in four patients with cerebellar ataxia (but was absent in >80 healthy and disease controls), was present at high titers, and mainly belonged to the IgG1 subclass.

## Samples and methods

### Samples

Anonymized testing of control samples as part of a study evaluating the sensitivity and specificity of an immunohistochemical assay for NMO-IgG [33] using mouse, rat and monkey cerebellum tissue sections led to the incidental identification of an anti-PC antibody in four serum samples from three patients with a binding pattern similar to that reported for anti-Ca [30]. The study was approved by the institutional review board of the medical faculty of the University of Heidelberg. The samples had originally been sent for testing of antibodies associated with autoimmune cerebellar ataxia. In the index case “cerebral ataxia” was explicitly mentioned as the patient’s diagnosis, in a second case, the diagnosis was that of “chronic cerebellar syndrome responsive to immunotherapy”. No additional clinical information is available due to anonymization. A further patient (female, age 28 years at onset) was identified by routine testing for cerebellar antibodies. This patient had suffered from progressive cerebellar ataxia since 2004. Symptoms included ataxia of the upper limbs, dysarthria, and gaze disturbances. MRI showed moderate cerebellar atrophy. Steroid treatment in 2007 and ten cycles of plasma exchange treatment in the same year had failed to halt disease progression. However, the patient’s symptoms have not worsened since 2010, and she was still able to work full-time in an office at last follow-up in June 2013. The patient and a relative tested positive for BCRA1, which is associated with increased risk of cancer, but extensive tumor screening (including CT, PET, mammography, and breast ultrasound) was negative. The patient gave written informed consent.

### Immunohistochemistry (IHC)

IHC was performed on cryosections of adult mouse, rat, and rhesus monkey cerebellum, rhesus monkey ocular bulb, and rhesus monkey intestine tissue (Euroimmun, Luebeck, Germany). Mouse, rat, and monkey tissue was provided unfixed as snap-frozen sections (4 to 6 μm) and was fixed with 10% formalin in phosphate-buffered saline (PBS) for 4 min before testing. For some experiments, 0.125% Triton X-100 or 1% 3-[(3-cholamidopropyl)dimethylammonio]-1-propanesulfonate in PBS was applied to the sections for 4 min. Sections were then washed in PBS, blocked with 10% goat serum or 10% donkey serum, depending on the secondary antibodies used, and, after three washes in chilled PBS, incubated with patient serum for 1 h or with various commercial antibodies for up to 2 h. Binding of human IgG, IgA, and IgM to central nervous system (CNS) tissue was detected by use of polyclonal goat anti-human IgG antibodies conjugated to fluorescein isothiocyanate (FITC) (Euroimmun), Alexa Fluor® (AF) 488 (Invitrogen, Karlsruhe, Germany) or AF568 (Invitrogen), polyclonal donkey anti-human IgG antibodies labeled with Rhodamin Red-X (Dianova, Hamburg, Germany), and polyclonal goat anti-human IgM and anti-human IgA antibodies conjugated to FITC (Euroimmun), respectively. For evaluation of IgG subclasses, unconjugated sheep anti-human IgG antibodies specific for IgG subclasses 1 to 4 (The Binding Site, Schwetzingen, Germany) were substituted for the FITC-labeled goat anti-human IgG antibody, and AF568-labeled donkey anti-sheep IgG (Invitrogen; absorbed against human IgG) was used to detect the subclass-specific antibodies. Binding of the following commercial antibodies was detected using goat anti-rabbit IgG AF568 (Invitrogen), goat anti-mouse IgG AF568 (Invitrogen), donkey anti-chicken IgG Rhodamin-Red X (Dianova), or donkey anti-goat IgG AF488 (Invitrogen) as secondary antibodies, depending on the primary antibodies employed and on further secondary antibodies used in double-labeling experiments: rabbit anti-inositol-triphosphate receptor type I (ITPR1) (Dianova); anti-Rho GTPase-activating protein 26 (ARHGAP26) (Santa Cruz, Heidelberg, Germany); goat anti-Homer3 (Santa Cruz); rabbit anti-protein kinase C gamma (PKCγ) (Santa Cruz); mouse anti-metabotropic glutamate receptor 1α (mGluR1α) (BD Pharmingen, Heidelberg, Germany); rabbit anti-glutamate receptor delta 2 (GluRδ2) (Santa Cruz); mouse anti-glutamate receptor 3 (GluR3, clone 3B3) (Millipore, Schwalbach, Germany); chicken anti-glial fibrillary acidic protein (GFAP) (Encor Biotechnology, Gainesville, FL, USA); rabbit anti-aquaporin4 (AQP4) (Sigma Aldrich, Taufkirchen, Germany); mouse anti-calbindin-D (Swant, Bellinzona, Switzerland). For selected experiments, patient serum was incubated with either full-length ITPR1 purified from rat cerebellum [34] or recombinant full-length human ARHGAP26 (Abnova, Taipei, Taiwan) overnight at 4°C prior to testing; the sera were then centrifuged at 11,000 rpm for 10 min and the supernatants incubated with brain tissue sections as described above. Sections were then mounted using glycerol standard immunofluorescence mounting medium containing 4’,6-diamidino-2-phenylindole (DAPI) (1:1,000) (Euroimmun) or ProLong Gold antifade reagent (Invitrogen). Slides were analyzed on a Nikon 90i and a Nikon Ni-E fluorescence microscope (Nikon Imaging Center, University of Heidelberg, Heidelberg, Germany).

### Protein array

A commercially available human protein microarray (Protoarray v5.0; Invitrogen) spotted with >9,000 human full-length proteins purified from a baculovirus-based expression system previously successfully employed to identify ARHGAP26 as the target antigen of anti-Ca was probed with the patient serum according to the manufacturer’s instructions as described [30].

### Dot-blot assay

Protran BA79 nitrocellulose membranes (0.1 μm; Whatman) were spotted with increasing dilutions (1.1.5, 1:3, 1:6, 1:12) of full-length ITPR1 purified from rat cerebellum (0.6 μg/μL solution; 9 μL/spot) [34] and of ARHGAP26 (0.14 μg/μL solution; 10 μL/spot; Abnova) in 0.1% bovine serum albumin (BSA). After drying, membranes were blocked with 5% BSA in Tris-buffered saline (TBS) for 1 h at room temperature, washed three times in TBS with 0.05% Tween (TBS-T), and finally incubated with a 1:20 dilution of the patient serum in 0.1% BSA/TBS-T for 1 h at room temperature. A donkey anti-human IgG antibody labeled with IRdye 700DX (Rockland) was used to detect bound IgG. Stripes were finally washed in TBS and analyzed using an Odyssey™ fluorescence scanner (Licor, Lincoln, NE, USA) and Odyssey™ 2.0.40 application software (Licor). As controls, serum samples from healthy donors were tested in the same run.

### Histoimmunoprecipitation (Histo-IP)

Cerebellum from rat or pig was dissected and shock-frozen in −160°C isopentane. The tissue was then cryosected (4 μm) with a SM2000R microtome (Leica Microsystems, Nussloch, Germany), placed on the entire surface of glass slides, and dried. Whole slides were then incubated with patient’s serum (diluted 1:100) at 4°C for 3 h followed by three washing steps with PBS containing 0.2% (w/v) Tween 20. Immunocomplexes were extracted from the sections by incubation in solubilization buffer (100 mmol/L Tris–HCl pH 7.4, 150 mmol/L sodium chloride, 2.5 mmol/L EDTA, 0.5% (w/v) deoxycholate, 1% (w/v) Triton X-100 containing protease inhibitors) at room temperature for 30 min. Detached material was homogenized and centrifuged at 16,000 × *g* at 4°C for 15 min. The clear supernatants were then incubated with Protein G Dynabeads (ThermoFisher Scientific, Dreieich, Germany) at 4°C overnight to capture immunocomplexes. The beads were then washed three times with PBS and eluted with PBS containing 5 mmol/L dithiothreitol and 1% (w/v) sodium dodecylsulfate at 95°C for 10 min, followed by SDS-PAGE and Western blot or mass spectrometry.

### SDS-PAGE and Western blot

Proteins were analyzed by SDS-PAGE using the NuPAGE system (ThermoFisher Scientific). Separated proteins were either identified by mass-spectrometric analysis or electrotransferred onto a nitrocellulose membrane by tank blotting with transfer buffer (ThermoFisher Scientific) according to the manufacturer’s instructions. The membranes were blocked with Universal Blot Buffer plus (Euroimmun) for 15 min and incubated with human serum or the polyclonal antibody against ITPR1 in Universal Blot Buffer plus for 3 h, followed by three washing steps with Universal Blot Buffer (Euroimmun), a second incubation for 30 min with anti-rabbit IgG-AP (Sigma-Aldrich), three washing steps, and staining with NBT/BCIP substrate (Euroimmun).

### Mass spectrometry

Mass spectrometry sample preparation was performed as reported by Koy et al. [35]. Unless otherwise indicated, hardware, software, MALDI targets, peptide standards, and matrix reagents were obtained from Bruker Daltonics, Bremen, Germany. Briefly, samples were reduced with dithiothreitol and carbamidomethylated with iodoacetamide prior to SDS-PAGE. Proteins were visualized with Coomassie Brilliant Blue G-250 and visible protein bands were excised and destained. After tryptic digest, peptides were extracted and spotted with α-cyano-4-hydroxycinnamic acid onto a MTP AnchorChip™ 384 TF target. MALDI-TOF/TOF measurements were performed with an Autoflex III smartbeam TOF/TOF200 System using flexControl 3.0 software. MS spectra for peptide mass fingerprinting (PMF) were recorded in positive ion reflector mode with 500 shots and in a mass range from 700 Da to 4,000 Da. Spectra were calibrated externally with the commercially available Peptide Calibration Standard II, processed with flexAnalysis 3.0, and peak lists were analyzed with BioTools 3.2. The Mascot search engine Mascot Server 2.3 (Matrix Science, London, UK) was used for protein identification by searching against the NCBI database limited to Mammalia. Search parameters were as follows: mass tolerance was set to 80 ppm, one missed cleavage site was accepted, and carbamidomethylation of cysteine residues as well as oxidation of methionine residues were set as fixed and variable modifications, respectively. To evaluate the protein hits, a significance threshold of *P* <0.05 was chosen. For further confirmation of the PMF hits, two peptides of each identified protein were selected for MS/MS measurements using the WARP feedback mechanism of BioTools. Parent and fragment masses were recorded with 400 and 1,000 shots, respectively. Spectra were processed and analyzed as described above with a fragment mass tolerance of 0.7 Da.

### Cloning and expression of ITPR1 in HEK293

The coding DNA for human ITPR1 (Genbank # BC172648, Source BioScience LifeSciences, Nottingham, UK) was transferred into the expression vector pTriEx-1 (Merck). The receptor was expressed in the human cell line HEK293 after ExGen500-mediated transfection (ThermoFisher Scientific) according to the manufacturer’s instructions. For the preparation of substrates for a recombinant cell-based indirect immunofluorescence assay (RC-IFA), HEK293 were grown on sterile cover glasses, transfected, and allowed to express the recombinant proteins for 48 h. Cover glasses were washed with PBS, fixed with acetone for 10 min at room temperature, air-dried, cut into millimeter-sized fragments (biochips), and used as substrates in RC-IFA. Alternatively, cells were transfected in standard T-flasks and the cells were harvested after 5 days. The cell suspension was centrifuged at 1,500 × *g*, 4°C for 20 min and the resulting sediment was extracted with solubilization buffer (see above). The extracts were stored in aliquots at −80°C until further use.

## Results

### Detection of a new Purkinje cell reactivity

**Figure 1:**
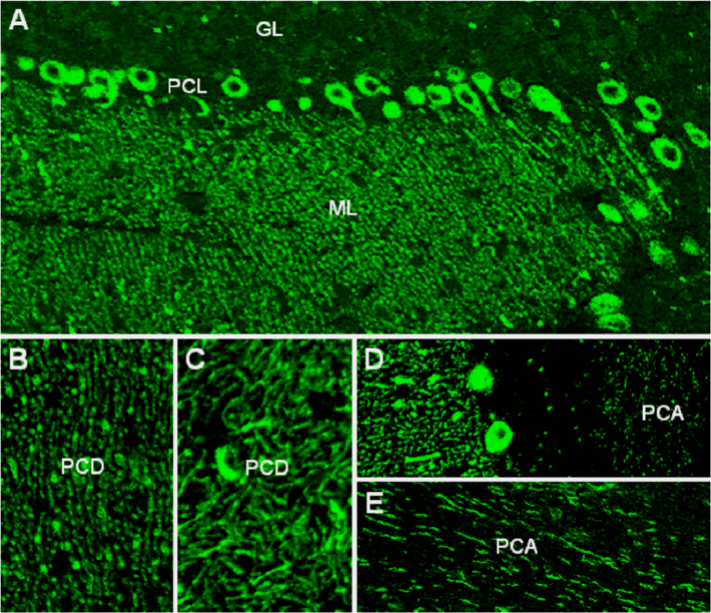
**Binding of patient IgG to the Purkinje cell (PC) dendrites (PCD) in the molecular layer (ML), to the PC somata in the PC layer (PCL), and to the PC axons (PCA) in the white matter (WM) on murine cerebellum tissue sections.** The observed staining pattern **(A)** is similar to that observed with anti-Ca/ARHGAP26 (see reference [30]). Note that PC nuclei, interneurons, and granular cells are spared. Also note that the PCD **(B and C)** and the PCA **(D and E)** staining patterns can differ significantly depending on sectional planes. Axonal staining may not be detectable on all sections. GL, Granular cell layer.

Immunohistochemistry on formalin-fixed frozen adult mouse, rat, and monkey tissue sections demonstrated strong reactivity of serum to structures in the molecular layer (ML), the Purkinje cell layer (PCL), and the white matter (WM) of the cerebellum (Figure 1), similar to that of anti-Ca/ARHGAP26 [30-32]. More detailed analysis at higher magnification revealed binding of IgG to somata, dendritic trunks, dendritic branches, and possibly dendritic spines of PCs, as previously described for anti-ARHGAP26 (anti-Ca). In addition, axons in the WM were stained by the patients’ serum. Double labeling with anti-GFAP and anti-AQP4 revealed no binding of the patient antibody to astrocytes in the WM and the granular layer (GL), or to Bergman glial cells in the PCL and ML (not shown). As anti-Ca/ARHGAP26, the antibodies spared interneurons, such as stellate cells, basket cells, and Golgi cells, as well as granular cells. The antibody stained PC somata and PC axons more intensely than anti-Ca/ARHGAP26-positive sera in a direct comparison, equaling the fluorescence intensity of the molecular layer. Interestingly, using primate enteric tissue sections, no binding to the plexus myentericus was observed, but there was strong staining of both the circular and the longitudinal tunica muscularis as well as of the muscularis propria and of blood vessel-related muscle cells, in addition to a fine, linear fluorescence adjacent to the epithelium of the intestinal villi. No such fluorescence was detected with anti-ARHGAP26-positive sera, which stained the myenteric plexus but not the muscle layers. Of note, enteric tissue sections have been previously used to differentiate between anti-Hu and anti-Ri antibodies, two anti-neuronal antibodies that are otherwise difficult to distinguish. We decided to refer to the specific staining pattern described here as anti-Sj throughout the manuscript, following a widely accepted convention to name newly described antibodies with reference to the index sample’s initials or code.

In a direct comparison of mouse, rat, and monkey cerebellum tissue sections, incubated simultaneously with the patients’ sera within the same well, mouse tissue was found to produce a slightly more clear and distinct signal than rat and primate tissue (mouse > rat > primate).

**Figure 2:**
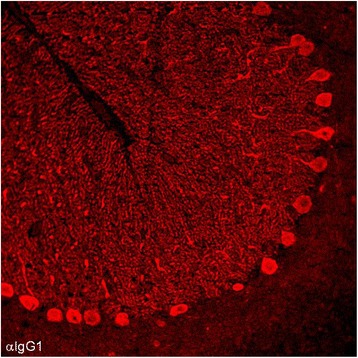
**Analysis of the IgG subclasses of anti-Sj in the index patient.** Subclass analysis revealed mainly IgG1 antibodies (depicted in red) with very few IgG2 and no IgG3 or IgG4 antibodies (not shown); no PC-specific antibodies to IgA or IgM were detectable (not shown). IgG1 was also the main anti-Sj IgG subclass in a second patient (not shown).

The immunoglobulin class and IgG subclass repertoire of the PC antibodies was analyzed by IHC in two patients—no material was left for analysis in the remainder—and revealed mainly IgG1 antibodies in both cases (Figure 2). Very weak IgG2 or IgG3 staining was observed in both patients and in one patient, respectively. In both patients, no anti-PC antibodies of the IgM or IgA class were detectable.

Cerebrospinal fluid was available from a single serum-positive patient and showed PC-specific staining identical to that observed with the patient’s serum sample.

### No evidence of previously described CNS autoantibodies

All reference sera were negative for anti-ARHGAP26 in a previously described dot-blot assay [30-32]. The index serum was, in addition, tested for anti-ARHGAP26 in the same commercial protein microarray originally used to identify ARHGAP26 as the target antigen of anti-Ca [30], which employs full-length human ARHGAP26 from a second, independent manufacturer (Invitrogen), but was also negative in this assay (median fluorescence units [FU] at 635 nm, 38; median fluorescence of all proteins, 158, compared to 55,323 and 181, respectively, in the anti-Ca/ARHGAP26 index case [30]). No evidence was found for anti-Hu, anti-Ri, anti-Yo, anti-Ma, anti-Ta, anti-CV2/CRMP5, anti-amphiphysin, ANNA-3 [14], PCA-2 [13], or anti-Tr/DNER [8,36,37]. Antibodies to Homer-3 [22], PKCγ [10], mGluR1[12,38], and GluRδ2 [20,21], which have been described in occasional patients with cerebellar ataxia and are known to bind to PC somata and/or dendrites, were ruled out by double-labeling experiments employing mouse, rat, and monkey cerebellum tissue sections as well as by Western blot analysis (not shown). Anti-CARPVIII [15,16] and Homer3 were excluded by RC-IFA (Euroimmun) and based on Western blotting. Antibodies to aquaporin-4, myelin oligodendrocyte glycoprotein, NMDA receptor, AMPA receptors 1 and 2, GABA B receptor, DPPX, LGI1, CASPR2, and Homer-3 were ruled out by IFA on cerebellum and hippocampus tissue sections and by RC-IFA using HEK293 expressing these antigens (Euroimmun). PKCγ, Zic4, GAD, amphiphysin, and GluRδ2 were included also in the protein microarray incubated with the index patient’s serum, but were not recognized by the patient’s IgG.

### Identification of ITPR1 as the target antigen by IFA and dot blot

**Figure 3:**
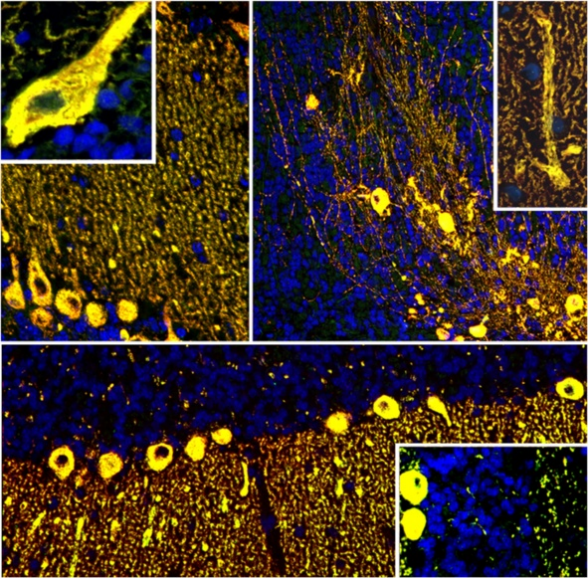
**Perfect overlay of the binding pattern observed with patient IgG and that observed with a commercial antibody to ITPR1, a well-established specific marker of Purkinje cells (PCs).** Anti-ITPR1 reactivity is depicted in red (Alexa Fluor® 568), the patient antibody in green (Alexa Fluor® 488), and yellow indicates overlay of the two antibodies. Nuclei are shown in blue (DAPI).

**Figure 4:**
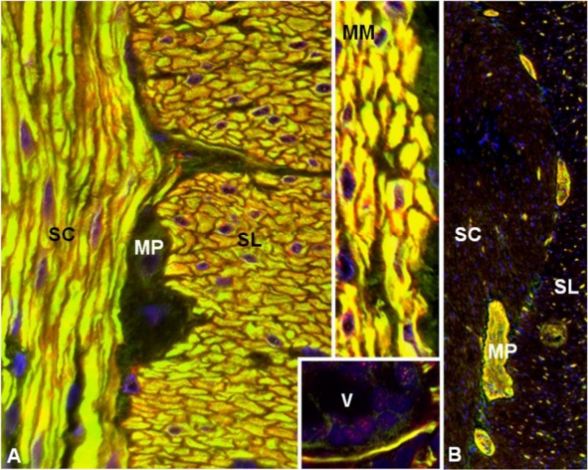
**Double labeling of primate intestine sections with patient serum and with commercial antibodies to anti-ITPR1 or ARHGAP26, respectively.** The anti-Sj index serum and the commercial antibody to ITPR1 **(A)** stained both the stratum circulare (SC) and the stratum longitudinale (SL) of the tunica muscularis as well as the muscularis mucosae (MM) and structures adjacent to the enteric villi (V), with a perfect overlay, but spared the plexus myentericus Auerbach (MP), which is located between SC and SL. Conversely, the anti-Ca index serum [30] and the commercial antibody to ARHGAP26 **(B)** both stained the MP (and the plexus submucosus Meissner; not shown) but spared the enteric muscle cells. Anti-ITPR1 or anti-ARHGAP26 reactivity is depicted in red (Alexa Fluor® 568), the patient antibody in green (Alexa Fluor® 488), and yellow indicates overlay of the two antibodies. Nuclei are shown in blue (DAPI).

**Figure 5:**
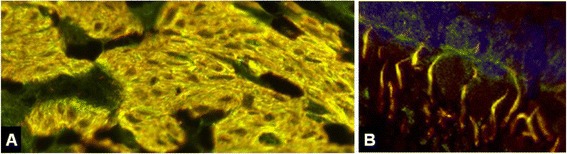
**Double labeling of primate bulbus oculi tissue sections with patient serum and a commercial antibody to anti-ITPR1.** An overlay of the index patient’s IgG and a commercial antibody to ITPR1 was also observed outside the CNS and the intestine, e.g., in the eye bulb (**A**: ciliary muscle, **B**: retina with rod and cone processes), confirming the specificity of the patient antibody for ITPR1. Anti-ITPR1 reactivity is depicted in red (Alexa Fluor® 568), the patient antibody in green (Alexa Fluor® 488), and yellow indicates overlay of the two antibodies. Nuclei are shown in blue (DAPI).

**Figure 6:**
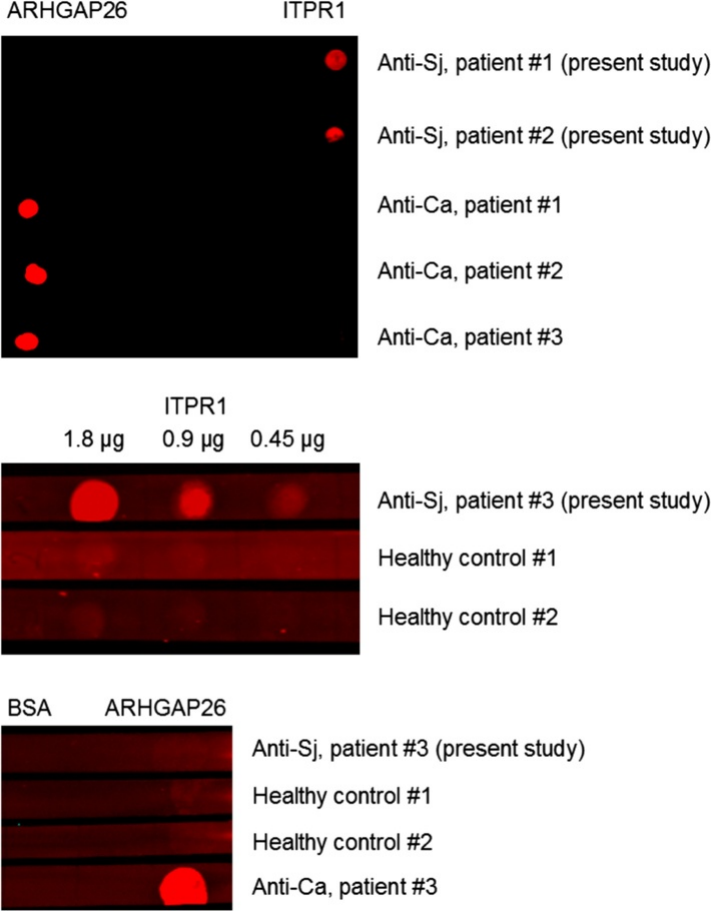
**Results from an dot-blot assay employing purified ITPR1 and ARHGAP26 as test substrates.** IgG from the patients’ serum bound to full-length ITPR1 but not to human full-length ARHGAP26; contrarywise, anti-Ca-positive control sera bound to ARHGAP26 but not to ITPR1 **(upper panel)**. The minimum amount of ITPR1 that resulted in a significant staining intensity was 0.45 μg/μL **(middle panel)**. Healthy control sera bound neither to ITPR1 nor to ARHGAP26 **(middle and lower panel)**.

The staining pattern observed with the patients’ serum highly resembled the pattern observed by us in a previous study with a commercial antibody to the inositol 1,4,5-trisphosphate receptor, type 1 (ITPR1) [30]. Double staining of cerebellum sections with that commercial antibody, which is used as a well-established marker of PCs in our laboratory, showed a perfect overlay with the staining pattern found with the patient antibody in the ML, PCL and WM (Figure 3). By contrast, sera positive for anti-ARHGAP26 had shown only partial overlay using the same commercial anti-ITRP1 antibody (see Figure 13 in ref. [30]). In addition, an overlay between the patients’ IgG and the commercial anti-ITPR1 antibody was also observed on other tissue sections, including intestine (Figure 4) and bulbus oculi sections (Figure 5), corroborating ITPR1 as the target antigen. In line with these findings, IgG from the patients’ sera but not from healthy controls bound to ITPR1 purified from rat cerebellum in a dot-blot assay (Figure 6).

### Confirmation of ITPR1 as the target antigen by competitive IHC

**Figure 7:**
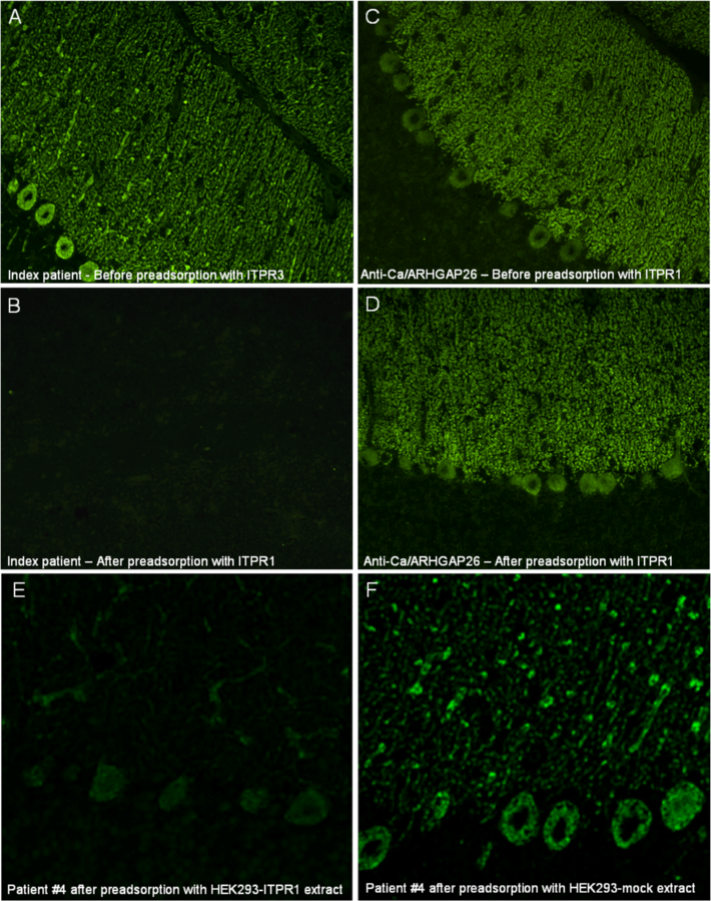
**Results from preadsorption experiments.** Preadsorption of the patient serum with purified rat ITPR1 or an extract of HEK293 cells expressing murine ITPR1 resulted in complete loss of binding to cerebellar tissue sections in an indirect immunofluorescence assay **(A, B, E)**. In contrast, binding of anti-Ca/ARHGAP26-positive sera was not affected by preadsorption with ITPR1 **(C, D)**. Preadsorption of anti-ITPR1-positive patient serum with full-length human ARHGAP26 (not shown) or with an extract of mock-transfected HEK293 **(F)** did not affect binding to PCs.

Preadsorption of the patient sera with rat ITPR1 protein resulted in complete loss of binding to cerebellum tissue sections (Figure 7); by contrast, preadsorption with ARHGAP26 did not. Interestingly, only preadsorption with full-length ITPR1 resulted in loss of PC staining, not preadsorption with a partial recombinant protein (2470 a.a. to 2577 a.a.; Abnova, Taiwan), indicating that the target epitope either depends on protein conformation or glycosylation or is located outside the residues 2470 a.a. to 2577 a.a.

### Parallel identification of ITPR1 as the target antigen by MALDI-TOF

**Figure 8:**
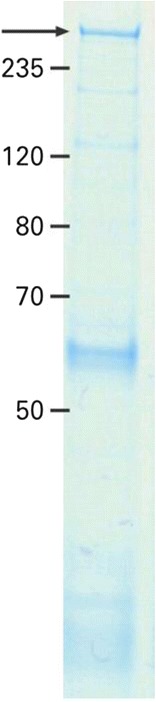
**Histoimmunoprecipitation with a reference patient serum revealed a band at around 300 kDa (arrow).**

Histoimmunoprecipitates from either rat or porcine cerebellum contained high amounts of IgG when one of the reference sera was used, whereas they were generally low after incubation of sera from healthy controls. Next to the immunoglobulins, the immunoprecipitated PC antibody-positive reference serum showed a protein band corresponding to a molecular mass of approximately 300 kDa in SDS-PAGE stained with colloidal Coomassie (Figure 8). The band was absent in the control samples. The proteins precipitating from rat and porcine cerebellum were identified as ITPR1 from the corresponding organisms by mass-spectrometric analysis. Western blot analysis with the polyclonal rabbit anti-ITPR1 antibody showed a strong reaction at 300 kDa of the immunoprecipitate obtained with the patient serum, while there were no reactions with fractions obtained with control sera. When used for double staining in IFA, the polyclonal anti-ITPR1 antibody produced an overlay with the reference serum used in the MALDI-TOF experiments.

### Confirmation of ITPR1 as the target antigen by recombinant cell-based IFA

**Figure 9:**
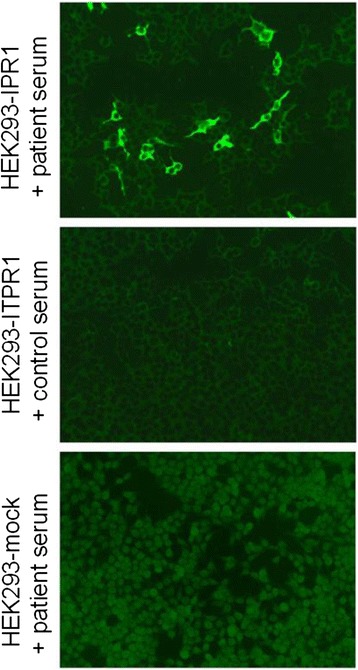
**Confirmation of ITPR1 as the target antigen by a recombinant cell-based indirect immunofluorescence assay employing HEK293 cells transfected with full-length human ITPR1 and mock-transfected HEK293 cells as control substrates.**

As further confirmation of correct antigen identification, the PC antibody-positive sera and controls were then analyzed by an RC-IFA using HEK293 expressing murine ITPR1 and mock-transfected HEK293 (Figure 9, upper panel). All reference sera but none of the controls reacted with the ITPR1-expressing cells (Figure 9, middle panel). In contrast, mock transfection did not result in any antibody binding (Figure 9, lower panel). The congruence of the patients’ autoantibody target and ITPR1 was further demonstrated by the dose-dependent competitive abolition of antibody binding to PCs by HEK293 lysates containing ITPR1 (Figure 7E). Antibody binding was unaffected when comparable lysates from mock-transfected HEK293 were used (Figure 7F).

### Specificity of anti-ITPR1 autoantibodies

Sera from 30 patients with various neural autoantibodies (anti-NMDAR, anti-Hu, anti-Yo, anti-Ri, anti-aquaporin 4, anti-LGI1, anti-CASPR2) and from 50 healthy controls were analyzed in the RC-IFA in parallel to the samples of the PC antibody-positive patients. None of the sera produced an immunofluorescence pattern similar to that of the index sera on the recombinant substrate. None of the four available anti-Ca/anti-ARHGAP26-positive sera [30-32] bound to full-length rat ITPR1 in the dot-blot assay or to full-length human ITPR1 in the RC-IFA.

## Discussion

Here we report on serum autoantibodies with high specificity to PCs. Using a broad panel of immunological methods, including immunohistochemistry and histoimmunoprecipitation combined with mass spectrometry, protein-based immunoassays, and RC-IFA, we identified ITPR1 as the target antigen of this serum reactivity. ITPR1 is an ligand-gated calcium channel that modulates intracellular calcium signaling following stimulation by inositol 1,4,5-trisphosphate [39,40].

Our findings expand the panel of diagnostic serum markers of autoimmune cerebellar ataxia. Due to the sometimes very similar tissue stainings, it is important to differentiate anti-ITPR1 from other PC autoantibodies in general, and particularly from anti-ARHGAP26 antibodies [30-32], by means of a specific assay.

Whether anti-ITPR1 is pathogenic is unknown. On the one hand, there is some indirect evidence for a potential pathogenic role: First, the antibody is highly specific for PCs, a cell type expressed exclusively in the cerebellum, and the patients presented with cerebellar ataxia. Second, the antibody belonged to the IgG1 subclass, which is known to be a strong complement activator, suggesting that it may act on PCs via complement-dependent mechanisms, which are well-established features in other autoantibody-associated disorders [41,42], though other direct effects such as antibody-dependent cell-mediated cytotoxicity or induction of apoptosis might also play a role. Third, the antibody was present at high titers (1:5,000, 1:3,200, 1:3,200, 1:1,000, according to IHC). Moreover, mutations of ITPR1 have been found in patients with spinocerebellar ataxia 15 (SCA15) and 29 (SCA29), drawing a link between malfunction of ITPR1 and disease [43,44]. SCA29 is an autosomal dominant disorder with onset in infancy characterized by a very slowly progressive or non-progressive gait and limb ataxia associated with cerebellar atrophy on brain imaging. Heterozygous mutations in the ITPR1 gene cause SCA15 with later onset. ITPR1-deficient mice born alive also show severe ataxia [45]. On the other hand, ITPR1 is primarily an intracellular antigen located in membranes encompassing the endoplasmatic or sarcoplasmatic (in muscle cells) reticulum, though surface localization has also been reported under certain circumstances [46-50]. Many authors believe that intracellular antigens are not accessible to antibodies *in vivo*. In fact, most neurological autoantibodies of proven pathogenic impact, such as antibodies to AQP4 in neuromyelitis optica [42,51-53], acetylcholine receptor in myasthenia gravis, VGCC in Lambert-Eaton syndrome [54], and mGluR1 in paraneoplastic cerebellar degeneration [11] target superstitial proteins of the plasma membrane. Moreover, passive transfer of antibodies to nuclear antigens such as anti-Yo [55-57] have not produced clinical disease in animal studies. Instead, T cell-mediated immune mechanisms directed against the target antigen of the accompanying antibody have been proposed to play a role in those disorders [58-61]. It is therefore possible that the antibody has diagnostic but no pathogenic impact, similar to the situation in many paraneoplastic neurological syndromes.

Autoantibody-associated cerebellar ataxia is frequently of paraneoplastic nature [1,2]. However, no data on most patients’ tumor status were available in this study. It will be of utmost importance to carefully examine future, prospectively identified patients with anti-ITPR1 antibodies for associated tumors. Of note, paraneoplastic antibodies and the associated syndromes can precede tumor diagnosis by several years. In a large study on patients with anti-Yo antibodies, the most common paraneoplastic serum reactivity associated with autoimmune cerebellar ataxia, the neurologic syndrome preceded the diagnosis of cancer by up to 15 months and in many cases led to that diagnosis [5]. This will make it crucial to follow-up future patients for at least 2 years. Of note, non-paraneoplastic ACA have been described as well, including ACA associated with antibodies to glutamate decarboxylase [23,24], tissue transglutaminase [18], GluRδ2 [20,21], and Homer-3 [22]. Anti-Ca/ARHGAP26-associated ACA has been reported both in a paraneoplastic context and in patients with no known tumor at the time of anti-ARHGAP26 testing [30-32].

We have so far identified four patients with ACA and anti-ARHGAP26 [30-32] and four with anti-ITPR1. Interestingly, several further samples sent to our laboratories with a diagnosis of ACA showed a staining pattern similar to that observed with anti-ARHGAP26 and anti-ITPR1, i.e., marked staining of the PC somata, the dendrites, and, partly, the axons, but did not react with either ARHGAP26 or ITPR1. Whether these sera contain antibodies other than anti-ARHGAP26 and anti-ITPR1, e.g., anti-CARPVIII or other novel autoantibodies, is currently under investigation.

## Conclusions

We describe a new autoantibody to PC somata, dendrites, and axons associated with cerebellar ataxia. The antibody targets ITPR1 and mainly belongs to the IgG1 subclass. Our findings indicate a possible role of autoimmunity to ITPR1 in the pathogenesis of autoimmune encephalitis and expand the panel of diagnostic markers for this condition.

## Abbreviations

ACA: Autoimmune cerebellar ataxia
AF: Alexa Fluor
ARHGAP26: Rho GTPase activating protein 26
BSA: Bovine serum albumin
CARPVIII: Carbonic anhydrase-related protein VIII
CNS: Central nervous system
DAPI: 4’,6-diamidino-2-phenylindole
DPPX: Dipeptidyl-peptidase 6
FITC: Fluorescein isothiocyanate
GFAP: Glial fibrillary acidic protein
GL: Granular layer
GluR3: Glutamate receptor 3
GluRδ2: Glutamate receptor delta 2
IFA: Indirect immunofluorescence
IgA: Immunoglobulin A
IgG: Immunoglobulin G
IgM: Immunoglobulin M
IHC: Immunohistochemistry
ITPR1: Inositol 1,4,5-trisphosphate receptor, type 1
mGluR1α: Metabotropic glutamate receptor 1α
ML: Molecular layer
MS: Mass spectrometry
PBS: Phosphate-buffered saline
PC: Purkinje cell
PCA: Purkinje cell antibody
PCL: PC layer
PKCγ: Protein kinase C gamma
PMF: Peptide mass fingerprinting
RC-IFA: Recombinant cell-based indirect immunofluorescence assay
SCA: Spinocerebellar ataxia
TBS: Tris-buffered saline
WM: White matter
